# Anhedonia in cocaine use disorder is associated with inflammatory gene expression

**DOI:** 10.1371/journal.pone.0207231

**Published:** 2018-11-08

**Authors:** Gabriel Rodrigo Fries, Sarwar Khan, Sydney Stamatovich, Elena Dyukova, Consuelo Walss-Bass, Scott D. Lane, Joy M. Schmitz, Margaret C. Wardle

**Affiliations:** Department of Psychiatry and Behavioral Science, University of Texas Health Science Center at Houston, Houston, TX, United States of America; University of Toronto, CANADA

## Abstract

Treatments for Cocaine Use Disorder (CUD) are variably effective, and there are no FDA-approved medications. One approach to developing new treatments for CUD may be to investigate and target poor prognostic signs. One such sign is anhedonia (i.e. a loss of pleasure or interest in *non-drug* rewards), which predicts worse outcomes in existing CUD treatments. Inflammation is thought to underlie anhedonia in many other disorders, but the relationship between anhedonia and inflammation has not been investigated in CUD. Therefore, we assessed peripheral genome-wide gene expression in n = 48 individuals with CUD with high (n = 24) vs. low (n = 24) levels of anhedonia, defined by a median split of self-reported anhedonia. Our hypothesis was that individuals with high anhedonia would show differential gene expression in inflammatory pathways. No individual genes were significantly different between the low and high anhedonia groups when using t-tests with a stringent false discovery rate correction (FDR-corrected *p* < 0.05). However, an exploratory analysis identified 166 *loci* where t-tests suggested group differences at a nominal *p* < 0.05. We used DAVID, a bioinformatics tool that provides functional interpretations of complex lists of genes, to examine representation of this gene list in known pathways. It confirmed that mechanisms related to immunity were the top significant associations with anhedonia in the sample. Further, the two top differentially expressed genes in our sample, *IRF1* and *GBP5*, both have primary inflammation and immune functions, and were significantly negatively correlated with total scores on our self-report of anhedonia across all 48 subjects. These results suggest that prioritizing development of anti-inflammatory medications for CUD may pay dividends, particularly in combination with treatment-matching strategies using either phenotypic measures of anhedonia or biomarkers of inflammatory gene expression to individualize treatment.

## Introduction

Cocaine use disorder (CUD) affects approximately 913,000 people aged 12 or older in the United States, and constitutes a substantial burden on the health care system [1,2]. Many treatments have been attempted for CUD, including psychosocial therapies and various pharmaceutical interventions. However, psychosocial interventions show variable efficacy, and to date, no pharmaceutical interventions are FDA approved. Thus, further investigation of the psychopathological manifestations and causes of CUD is needed to develop more effective therapies. One approach may be to identify symptoms associated with poor treatment outcomes, and investigate the mechanisms underlying these symptoms. A better understanding of the biological and psychological underpinnings of these poor prognostic signs may critically assist the development of novel effective treatments for CUD.

One potential key symptom in addictive disorders in general, and CUD in particular, is anhedonia. Anhedonia is defined as a decrease in pleasure capacity; here we use it to refer more specifically to a loss of pleasure or interest in *non-drug* rewards. Anhedonia is common in addictive disorders, including cocaine, but also alcohol, opiate, amphetamine, and cannabis disorders [3,4]. Across substances of abuse, the presence of anhedonia correlates with withdrawal symptom severity, craving, and likelihood of relapse, suggesting anhedonia may predict a more difficult course of addiction and worse treatment outcomes [3]. Several studies support the hypothesis that anhedonia is a poor prognostic sign in substance use disorders. For example, lifetime presence of anhedonia significantly predicts continued smoking during smoking cessation treatment, even after adjusting for the potential impact of co-occurring depressed mood [5]. Another study found that opiate dependent individuals who were less aroused by pleasant, non-drug related pictures were more likely to continue using heroin during treatment [6]. Most pertinent to CUD, low positive mood, measured using the Profile of Mood States (which could represent anhedonia), and the presence of anhedonia at treatment outset, as assessed using the Snaith-Hamilton Pleasure Scale, have been associated with poorer outcomes in CUD treatment [7,8]. Therefore, understanding and addressing the pathological basis of anhedonia in CUD may help produce more effective treatments for CUD.

A significant body of literature suggests that inflammation, or excessive immune activity, may underlie anhedonia across disorders [9–11]. During acute inflammatory events, such as a cold, circulating inflammatory mediators produce “sickness behaviors” such as reduced activity, which serve the adaptive function of conserving energy and speeding healing [11]. However, maladaptive chronic low-grade inflammation appears to be present in many psychiatric disorders, and has been implicated in symptoms of anhedonia seen in depression, schizophrenia, and cancer-related fatigue, among other conditions [9,12–14].

The neurological and biological mechanisms of anhedonia in CUD are not known, but one possibility is that cocaine use creates inflammation, which produces anhedonia. Some literature indicates inflammation is elevated in cocaine users, although findings are not fully consistent. In one study comparing crack cocaine users to healthy controls, users showed higher levels of the inflammatory mediators IL-1β, TNFα, and IL-10 [15]. A similar study indicated increases in IL-6 in cocaine users compared to healthy controls, although IL-10 was decreased in cocaine users [16]. Female crack cocaine users at the outset of treatment showed higher levels of IL-10, IL-4, and IL-6, which gradually moved closer to reference values throughout detoxification, indicating inflammation declines in conjunction with cocaine use [17]. A similar study of cocaine-using adolescents also found elevated IL-6 and IL-10 at treatment admission; however, in this study values did not change over treatment [18]. A study examining stress responses in cocaine users showed decreased basal IL-10 but enhanced TNFα responses to stress compared to social drinking controls [19]. However, other studies have either shown no differences in inflammatory biomarkers between individuals with CUD and controls [20], only shown differences in individuals with additional risk factors such as early life trauma [21], or even suggested immunosuppression in cocaine users [22]. Taken together, these mixed results suggest inflammation may be present only in a subset of cocaine users. We hypothesize these may be the same individuals who show anhedonia and more difficult clinical courses.

This study assessed peripheral genome-wide gene expression in individuals with CUD who were also displaying high levels of anhedonia, compared to individuals with CUD and low anhedonic symptoms. We examined gene expression in peripheral blood mononuclear cells, with the hypothesis that individuals with CUD and high levels of anhedonia would show different expression of genes in inflammatory pathways, compared to individuals with CUD and low levels of anhedonia. We assessed genome-wide expression for several reasons. First, gene expression shows promise as a peripheral biomarker of alterations in brain functioning, with studies suggesting that peripheral changes in expression, particularly in inflammatory pathways, relate to changes in brain tissue [23]. Further, a previous study in individuals with alcohol abuse discovered changes in the immune signaling pathways of alcohol abusers compared to healthy controls, suggesting this technique is sensitive to inflammatory changes in addiction [24]. We selected genome-wide analysis coupled with a bioinformatics approach because, as a complex condition, it is likely gene expression alterations in CUD are not limited to a few candidate genes, but rather to complex gene networks and potentially the accumulation of several unfavorable changes. Although the inflammation-anhedonia hypothesis has a strong theoretical basis, this exploratory approach was judged appropriate for an initial hypothesis-generating study that is the first to examine the biological underpinnings of anhedonia in CUD.

## Materials and methods

### Participants

Participants were 48 individuals with Cocaine Dependence per DSM-IV or CUD of at least moderate severity per DSM-5 (this study bridged the transition from DSM-IV to DSM-5) attending screening sessions used to determine eligibility for both treatment and non-treatment studies at a center focused on treatment development for addictions. All participants provided separate written informed consent for data collection during the screening, including the blood draw and gene expression analyses, and all procedures were carried out in accordance with the Declaration of Helsinki and with the approval of the local Institutional Review Board at the University of Texas Health Science Center at Houston. Screening consisted of a Structured Clinical Interview for DSM-IV or DSM-5 [25,26] and Addiction Severity Index–Lite [27] administered by a master’s level clinician, a physical exam conducted by a physician, an electrocardiogram, laboratory tests, and completion of several self-report forms, including self-report of anhedonia. Criteria for inclusion in the gene expression study were: 1. No major inflammatory/medical conditions (e.g. tuberculosis, Hepatitis C, HIV, diabetes, active infection, cancer) with the exception of hypertension or high cholesterol; 2. No psychoactive or daily medication use, except for hypertension or high cholesterol medications; 3. No psychotic disorders; 4. If female, not pregnant, breastfeeding, peri- or post-menopausal, having a hysterectomy, or taking oral contraceptives. Participants with other psychiatric or substance use disorders were included, as significant comorbidity is typical in this population.

### Self-report of anhedonia

Our measure of anhedonia was the Snaith-Hamilton Pleasure Scale (SHAPS), which assesses ability to enjoy non-drug rewards [28]. The SHAPS is a 14-item, self-report scale that asks participants to indicate on a scale from 0 = "Strongly Agree" to 3 = "Strongly Disagree" their ability to enjoy 14 normally pleasurable events (“I would enjoy my favorite television or radio program” or “I would get pleasure from helping others”). Scores range from 0 to 42 with higher scores indicating greater anhedonia. The SHAPS is one of the most widely used self-reports of anhedonia, with good psychometric characteristics [29], and previous validation in a substance dependent population [30]. A median split of SHAPS scores was used to create high and low anhedonia groups for the gene expression analysis.

### Genome-wide expression analysis

Peripheral blood was collected from fasting participants in the morning (between 8am-12pm), by venipuncture into Heparin-containing vacutainers. This was followed by isolation of peripheral blood mononuclear cells with Ficoll-Paque (GE Healthcare, Little Chalfont, UK) density gradient centrifugation, as described by the manufacturer. Isolated cells (up to 3 x 10^6^ per vial) were resuspended in RPMI 1640 medium (Thermo Fisher Scientific, Waltham, MA) containing 5% dimethyl sulfoxide (Sigma-Aldrich, St. Louis, MO) and stored at -80°C until further processed. RNA isolation was performed with the RNeasy Plus Mini Kit (Qiagen, Hilden, Germany), according to the manufacturer’s instructions. RNA quantification was performed on NanoDrop (Thermo Fisher Scientific) and the integrity of RNA samples was confirmed by assessing the RNA Integrity Number (RIN) using the RNA 6000 Nano Kit (Agilent, Santa Clara, CA) on Bioanalyzer (Agilent). Total RNA samples were converted into biotin-labeled cRNAs with the TargetAmp Nano Labeling Kit (Epicentre, Madison, WI), quantified on NanoDrop, and hybridized (750ng) to the HumanHT-12 v4 Expression BeadChip (Illumina, San Diego, CA), according to the manufacturer’s instructions. BeadChips were scanned on an iScan microarray reader (Illumina) immediately after the hybridization protocol. Raw scan data were uploaded into the GenomeStudio software v2011.1 (Illumina), where they were initially background subtracted and quantile normalized using the Gene Expression Module v1.0. Microarray data has been deposited into the NCBI GEO database, accession number GSE116833.

### Real-time quantitative PCR

The top two differentially expressed genes between groups were selected for validation using real time quantitative PCR. Briefly, RNA samples (160 ng) were initially converted into cDNA using the High Capacity cDNA Synthesis Kit (Life Technologies, Carlsbad, CA) and later diluted 5 times for the PCR reactions. Amplifications of Interferon Regulatory Factor 1 (*IRF1*) and Guanylate Binding Protein 5 (*GBP5*) were performed in 12 μL-reactions using inventoried FAM-MGB-labeled TaqMan Gene Expression Assays (Hs00971965_m1 and Hs00369472_m1 for *IRF1* and *GBP5*, respectively) and the VIC-MGB_PL-labelled beta-2-microglobulin (*B2M*) as endogenous control (Hs00187842_m1). PCR reactions were run on a QuantStudio 7 Flex Real-Time PCR System (Life Technologies) with each sample assayed in triplicate. Data were analyzed by the 2(-Delta Delta C(T)) method [31].

### Statistical analysis

Between-group *t*-tests and chi-squared tests with a significance level of *p* < 0.05 were used to evaluate for possible group differences that might represent confounds. Genome-wide expression levels were compared between low and high anhedonia groups using GenomeStudio software v2011.1 (Illumina). Differential expression analysis between low and high anhedonia groups was performed using the low anhedonia as the reference group, ‘Illumina custom’ as the error model, and multiple testing correction using the Benjamini and Hochberg procedure to control for FDR. In addition to this stringent analysis, an exploratory analysis was performed to identify differentially expressed genes with nominal *p*-values < 0.05. After checking for the normality of data on IBM SPSS Statistics 25 using Shapiro-Wilk’s test and histogram visualization, we also investigated the correlation between the top-ranked differentially expressed genes and total SHAPS scores with the Spearman rank correlation coefficient. Differentially expressed genes were analyzed for their collective functional annotation on Database for Annotation, Visualization and Integrated Discovery (DAVID) version 6.8 [32]. The enriched functional annotation terms were selected based on a false discovery rate (FDR) cutoff < 0.05.

## Results

Demographic data from CUD patients by group are shown in Table 1. Groups did not significantly differ on any demographic, substance use or medical/psychiatric variables tested (p > 0.05 for all comparisons). Race was collapsed into African-American vs. all others due to the small number of participants in other racial categories. In our sample the overall mean for the SHAPS was 11.68 (SD = 7.67), and the median split was at 11.5. This is similar to previous reports in individuals with substance use disorders [30], which tend to be above those of healthy adults, but below individuals with depression. Twenty seven percent of the sample met a previously established clinical cutoff for the presence of significant anhedonia on the SHAPS [28].

**Table 1:** Demographic variables by group.

Variables	Low Anhedonia Group (N = 24) M (SD) or N (%)	High Anhedonia Group (N = 24) M (SD) or N (%)	Statistic
Demographic			
Female gender	5 (21%)	6 (25%)	χ^2^(1) = 0.12, *p* = 0.73
Age	44.12 (8.87)	47.50 (8.53)	t(46) = -1.34, *p* = 0.19
African-American race	17 (71%)	20 (83%)	χ^2^(1) = 1.06, *p* = 0.30
Years of education	13.54 (1.84)	12.75 (1.89)	t(46) = 1.47, *p* = 0.15
Substance Use			
# of days cocaine use past month	15.17 (9.12)	16.04 (8.98)	t(46) = -0.34, *p* = 0.74
# of years using cocaine	14.42 (10.61)	16.17 (9.30)	t(46) = -0.61, *p* = 0.55
Cocaine+ UDS day of draw	16 Yes (67%)	19 Yes (79%)	χ^2^(1) = 0.95, *p* = 0.33
Other Current SUD diagnosis	7 Yes (29%)	9 Yes (38%)	χ^2^(1) = 0.38, *p* = 0.54
Medical/Psychiatric			
Hypertension	4 (17%)	4 (17%)	χ^2^(1) = 0.00, *p* = 1.00
Cholesterol mg/dl	165.58 (30.10)	182.92 (46.66)	t(46) = -1.48, *p* = 0.15
Fasting glucose mg/dl	85.21 (9.95)	84.04 (8.19)	t(46) = 0.44, *p* = 0.66
Regular use of medications	9 (38%)	7 (29%)	χ^2^(1) = 0.38, *p* = 0.54
Other psychiatric diagnosis	6 (25%)	7 (29%)	χ^2^(1) = 0.11, *p* = 0.75

Cocaine + UDS–Cocaine positive urine drug screen; SUD–Substance Use Disorder (including Alcohol Use Disorder); mg/dl–milligrams per deciliter.

No gene was significantly different between groups after correction for multiple testing (FDR-corrected *p* > 0.05 for all comparisons). We found 166 differentially expressed genes or putative *loci* between groups in the exploratory analysis (nominal *p* < 0.05), of which 41 were up-regulated and 125 were down-regulated in the high anhedonia group (Table 2).

**Table 2:** Differentially expressed genes in peripheral blood mononuclear cells from cocaine use disorder patients with low and high anhedonia (ranked by nominal *p*-value).

SYMBOL	Low anhedonia AVG	High anhedonia AVG	Nominal *p*-value	Fold change	Definition
*IRF1*	1857.4	1365.6	0.00221	0.735	Homo sapiens interferon regulatory factor 1 (IRF1), mRNA.
*GBP5*	1246.3	798.6	0.00288	0.640	Homo sapiens guanylate binding protein 5 (GBP5), mRNA.
*KCTD10*	162.4	115.3	0.00479	0.709	Homo sapiens potassium channel tetramerisation domain containing 10 (KCTD10), mRNA.
*PF4V1*	98.5	226.1	0.00506	2.295	Homo sapiens platelet factor 4 variant 1 (PF4V1), mRNA.
*C1orf71*	774.6	1000.9	0.00529	1.292	Homo sapiens chromosome 1 open reading frame 71 (C1orf71), mRNA.
*PID1*	199.9	304.7	0.00673	1.524	Homo sapiens phosphotyrosine interaction domain containing 1 (PID1), mRNA.
*NLRC5*	92.4	69.6	0.00766	0.753	Homo sapiens NLR family, CARD domain containing 5 (NLRC5), mRNA.
*SRXN1*	480.8	377.7	0.0082	0.785	Homo sapiens sulfiredoxin 1 homolog (S. cerevisiae) (SRXN1), mRNA.
*CXCL2*	541.9	235.1	0.00897	0.433	Homo sapiens chemokine (C-X-C motif) ligand 2 (CXCL2), mRNA.
*SLC40A1*	428.3	555.8	0.01012	1.297	Homo sapiens solute carrier family 40 (iron-regulated transporter), member 1 (SLC40A1), mRNA.
*PPP1R16B*	620.9	499.1	0.0111	0.803	Homo sapiens protein phosphatase 1, regulatory (inhibitor) subunit 16B (PPP1R16B), mRNA.
*PDIA4*	46.6	31.9	0.01143	0.684	Homo sapiens protein disulfide isomerase family A, member 4 (PDIA4), mRNA.
*C1orf21*	43.3	23.1	0.01242	0.533	Homo sapiens chromosome 1 open reading frame 21 (C1orf21), mRNA.
*LAP3*	1230.5	920.8	0.01302	0.748	Homo sapiens leucine aminopeptidase 3 (LAP3), mRNA.
*LOC441408*	94.4	73	0.01321	0.773	PREDICTED: Homo sapiens hypothetical LOC441408, transcript variant 1 (LOC441408), mRNA.
*TMEM170A*	161.8	127.6	0.01329	0.788	Homo sapiens transmembrane protein 170A (TMEM170A), mRNA.
*PRIC285*	761.8	556.4	0.01379	0.730	Homo sapiens peroxisomal proliferator-activated receptor A interacting complex 285 (PRIC285), transcript variant 2, mRNA.
*FCHSD2*	231.3	188.6	0.01382	0.815	Homo sapiens FCH and double SH3 domains 2 (FCHSD2), mRNA.
*PRC1*	71.5	51.6	0.01388	0.721	Homo sapiens protein regulator of cytokinesis 1 (PRC1), transcript variant 2, mRNA.
*SH3BGRL2*	285	425	0.01453	1.49	Homo sapiens SH3 domain binding glutamic acid-rich protein like 2 (SH3BGRL2), mRNA.
*CD55*	284.9	226.8	0.01486	0.796	Homo sapiens CD55 molecule, decay accelerating factor for complement (Cromer blood group) (CD55), mRNA.
*PDK4*	228.5	371.1	0.01529	1.624	Homo sapiens pyruvate dehydrogenase kinase, isozyme 4 (PDK4), mRNA.
*FLJ33590*	87.2	58.4	0.0153	0.669	Homo sapiens hypothetical protein FLJ33590 (FLJ33590), mRNA.
*SNCA*	208.6	277.4	0.01562	1.329	Homo sapiens synuclein, alpha (non A4 component of amyloid precursor) (SNCA), transcript variant NACP112, mRNA.
*SPATS2L*	177.6	122	0.0163	0.686	Homo sapiens spermatogenesis associated, serine-rich 2-like (SPATS2L), transcript variant 2, mRNA.
*STAT1*	2174	1456.3	0.01725	0.669	Homo sapiens signal transducer and activator of transcription 1, 91kDa (STAT1), transcript variant alpha, mRNA.
*TGFBR3*	1661.2	1222.6	0.01767	0.735	Homo sapiens transforming growth factor, beta receptor III (TGFBR3), mRNA.
*ARL4C*	208.3	163.1	0.01783	0.783	Homo sapiens ADP-ribosylation factor-like 4C (ARL4C), mRNA.
*RIPK2*	1123.6	846.9	0.01787	0.753	Homo sapiens receptor-interacting serine-threonine kinase 2 (RIPK2), mRNA.
*IFITM3*	3464.5	1863.3	0.01793	0.537	Homo sapiens interferon induced transmembrane protein 3 (1-8U) (IFITM3), mRNA.
*DUSP5*	1234.6	950.5	0.01852	0.769	Homo sapiens dual specificity phosphatase 5 (DUSP5), mRNA.
*C17orf97*	48.9	88.5	0.01864	1.809	Homo sapiens chromosome 17 open reading frame 97 (C17orf97), mRNA.
*CYLN2*	52.7	73	0.01948	1.385	Homo sapiens cytoplasmic linker 2 (CYLN2), transcript variant 2, mRNA.
*RGS18*	1318.6	1720.1	0.01965	1.304	Homo sapiens regulator of G-protein signaling 18 (RGS18), mRNA.
*BCAT1*	61.4	86.3	0.02051	1.405	Homo sapiens branched chain aminotransferase 1, cytosolic (BCAT1), mRNA.
*TAP1*	3242.5	2637.6	0.0215	0.813	Homo sapiens transporter 1, ATP-binding cassette, sub-family B (MDR/TAP) (TAP1), mRNA.
*HERC5*	955.6	644.8	0.02163	0.674	Homo sapiens hect domain and RLD 5 (HERC5), mRNA.
*APOBEC3H*	51.9	30.8	0.02165	0.593	Homo sapiens apolipoprotein B mRNA editing enzyme, catalytic polypeptide-like 3H (APOBEC3H), mRNA.
*KLF5*	24.2	11.1	0.02191	0.458	Homo sapiens Kruppel-like factor 5 (intestinal) (KLF5), mRNA.
*LBA1*	311.2	255.4	0.02252	0.820	Homo sapiens lupus brain antigen 1 (LBA1), mRNA.
*ZMYND15*	94.3	68.5	0.02335	0.726	Homo sapiens zinc finger, MYND-type containing 15 (ZMYND15), transcript variant 2, mRNA.
*HERC6*	295.6	215.7	0.02347	0.729	Homo sapiens hect domain and RLD 6 (HERC6), transcript variant 1, mRNA.
*PFN4*	4	14.4	0.02409	3.6	Homo sapiens profilin family, member 4 (PFN4), mRNA.
*KIAA1600*	473.5	393.3	0.02432	0.830	Homo sapiens KIAA1600 (KIAA1600), mRNA.
*RGS1*	643.7	370.8	0.02467	0.576	Homo sapiens regulator of G-protein signaling 1 (RGS1), mRNA.
*SLC25A28*	1486.8	1259.1	0.02474	0.846	Homo sapiens solute carrier family 25, member 28 (SLC25A28), mRNA.
*NAPB*	134.8	109.6	0.0249	0.813	Homo sapiens N-ethylmaleimide-sensitive factor attachment protein, beta (NAPB), mRNA.
*C20orf3*	718.3	606.4	0.02518	0.844	Homo sapiens chromosome 20 open reading frame 3 (C20orf3), mRNA.
*USP18*	44	16.8	0.02551	0.3818	Homo sapiens ubiquitin specific peptidase 18 (USP18), mRNA.
*NCOA7*	853.7	674.2	0.02568	0.789	Homo sapiens nuclear receptor coactivator 7 (NCOA7), mRNA.
*MYBPC3*	43.6	30.2	0.02598	0.692	Homo sapiens myosin binding protein C, cardiac (MYBPC3), mRNA.
*DGKE*	25.8	14.5	0.02606	0.562	Homo sapiens diacylglycerol kinase, epsilon 64kDa (DGKE), mRNA.
*LOC100132503*	68.6	88.3	0.02656	1.287	PREDICTED: Homo sapiens similar to ring finger protein 208 (LOC100132503), mRNA.
*LOC400759*	126.3	70	0.02685	0.554	Homo sapiens similar to Interferon-induced guanylate-binding protein 1 (GTP-binding protein 1) (Guanine nucleotide-binding protein 1) (HuGBP-1) (LOC400759) on chromosome 1.
*LOC197135*	201.7	128.4	0.02699	0.636	PREDICTED: Homo sapiens hypothetical LOC197135, transcript variant 5 (LOC197135), mRNA.
*REC8*	251.1	194.2	0.02707	0.773	Homo sapiens REC8 homolog (yeast) (REC8), transcript variant 1, mRNA.
*NFIL3*	946	707.4	0.02725	0.747	Homo sapiens nuclear factor, interleukin 3 regulated (NFIL3), mRNA.
*LOC729580*	88.3	69	0.02736	0.781	PREDICTED: Homo sapiens hypothetical LOC729580 (LOC729580), mRNA.
*C16orf33*	389.5	327.8	0.02783	0.841	Homo sapiens chromosome 16 open reading frame 33 (C16orf33), mRNA.
*CD8A*	2691	2060.5	0.02813	0.765	Homo sapiens CD8a molecule (CD8A), transcript variant 2, mRNA.
*LRRC57*	122.4	99.2	0.02817	0.810	Homo sapiens leucine rich repeat containing 57 (LRRC57), mRNA.
*CD83*	1912	1345.2	0.0283	0.703	Homo sapiens CD83 molecule (CD83), transcript variant 1, mRNA.
	673.5	556.7	0.02839	0.826	full-length cDNA clone CS0CAP005YH21 of Thymus of Homo sapiens (human)
	248	194.4	0.02872	0.783	Homo sapiens cDNA clone IMAGE:5277162
*F13A1*	659.8	875	0.02876	1.326	Homo sapiens coagulation factor XIII, A1 polypeptide (F13A1), mRNA.
*NECAP1*	563.1	473.3	0.02878	0.840	Homo sapiens NECAP endocytosis associated 1 (NECAP1), mRNA.
	12.1	23.6	0.02931	1.950	Human tissue plasminogen activator mRNA, partial cds
	31.1	19.7	0.02958	0.633	Homo sapiens cDNA FLJ35432 fis, clone SMINT2002311
*LOC648470*	345.6	281.4	0.02995	0.814	PREDICTED: Homo sapiens similar to Caspase-4 precursor (CASP-4) (ICH-2 protease) (TX protease) (ICE(rel)-II) (LOC648470), mRNA.
*GBP4*	760.3	544.7	0.03006	0.716	Homo sapiens guanylate binding protein 4 (GBP4), mRNA.
*EPSTI1*	1084.5	642.8	0.03101	0.592	Homo sapiens epithelial stromal interaction 1 (breast) (EPSTI1), transcript variant 2, mRNA.
*FAM50B*	52.2	72.4	0.03109	1.386	Homo sapiens family with sequence similarity 50, member B (FAM50B), mRNA.
*HSH2D*	257.2	202.8	0.03133	0.788	Homo sapiens hematopoietic SH2 domain containing (HSH2D), mRNA.
*LOC644590*	328.2	276.6	0.03143	0.842	PREDICTED: Homo sapiens similar to EVIN1 (LOC644590), mRNA.
*PSME2*	2178.8	1812.5	0.03168	0.831	Homo sapiens proteasome (prosome, macropain) activator subunit 2 (PA28 beta) (PSME2), mRNA.
*LOC649864*	54.2	39.7	0.03174	0.732	PREDICTED: Homo sapiens similar to HLA class I histocompatibility antigen, A-29 alpha chain precursor (MHC class I antigen A*29) (Aw-19), transcript variant 1 (LOC649864), mRNA.
*KDELC2*	146.1	119.6	0.03215	0.818	Homo sapiens KDEL (Lys-Asp-Glu-Leu) containing 2 (KDELC2), mRNA.
*UAP1*	293	245.3	0.03287	0.837	Homo sapiens UDP-N-acteylglucosamine pyrophosphorylase 1 (UAP1), mRNA.
*CLCF1*	102.4	80.9	0.03289	0.790	Homo sapiens cardiotrophin-like cytokine factor 1 (CLCF1), transcript variant 1, mRNA.
*IBSP*	9.4	19.9	0.03293	2.117	Homo sapiens integrin-binding sialoprotein (bone sialoprotein, bone sialoprotein II) (IBSP), mRNA.
*C9orf82*	97.9	78.6	0.03309	0.802	Homo sapiens chromosome 9 open reading frame 82 (C9orf82), mRNA.
*PGA5*	5.4	19.8	0.03319	3.666	Homo sapiens pepsinogen 5, group I (pepsinogen A) (PGA5), mRNA.
*ITM2C*	776.2	638.7	0.03364	0.822	Homo sapiens integral membrane protein 2C (ITM2C), transcript variant 2, mRNA.
*TRIM22*	976.5	775.9	0.03373	0.794	Homo sapiens tripartite motif-containing 22 (TRIM22), mRNA.
*SNORD114-2*	24.1	35.6	0.03385	1.477	Homo sapiens small nucleolar RNA, C/D box 114–2 (SNORD114-2), small nucleolar RNA.
*LAPTM4B*	67.1	89	0.03442	1.326	Homo sapiens lysosomal protein transmembrane 4 beta (LAPTM4B), mRNA.
*FAM46C*	1498.2	1164.3	0.03446	0.777	Homo sapiens family with sequence similarity 46, member C (FAM46C), mRNA.
*SLAMF7*	138.5	104	0.03478	0.750	Homo sapiens SLAM family member 7 (SLAMF7), mRNA.
*KBTBD8*	200.4	163.8	0.03491	0.817	Homo sapiens kelch repeat and BTB (POZ) domain containing 8 (KBTBD8), mRNA.
*IRF4*	185.1	152.3	0.03537	0.822	Homo sapiens interferon regulatory factor 4 (IRF4), mRNA.
*FFAR2*	63.5	35.4	0.03551	0.557	Homo sapiens free fatty acid receptor 2 (FFAR2), mRNA.
*TDRD9*	47.7	66.9	0.03554	1.402	Homo sapiens tudor domain containing 9 (TDRD9), mRNA.
*FBXO6*	295.5	232	0.03571	0.785	Homo sapiens F-box protein 6 (FBXO6), mRNA.
*IL12A*	34.6	21.7	0.03571	0.627	Homo sapiens interleukin 12A (natural killer cell stimulatory factor 1, cytotoxic lymphocyte maturation factor 1, p35) (IL12A), mRNA.
*GALM*	148.3	120.8	0.036	0.814	Homo sapiens galactose mutarotase (aldose 1-epimerase) (GALM), mRNA.
*PATL2*	182	116.5	0.03614	0.640	PREDICTED: Homo sapiens misc_RNA (PATL2), miscRNA.
*IFI44*	973.5	556.6	0.03638	0.571	Homo sapiens interferon-induced protein 44 (IFI44), mRNA.
*TNFRSF21*	156.3	111.6	0.03707	0.714	Homo sapiens tumor necrosis factor receptor superfamily, member 21 (TNFRSF21), mRNA.
*GNG11*	899.5	1303.2	0.03713	1.448	Homo sapiens guanine nucleotide binding protein (G protein), gamma 11 (GNG11), mRNA.
*TMEM88*	43.6	27.2	0.03735	0.623	Homo sapiens transmembrane protein 88 (TMEM88), mRNA.
*NRGN*	1413	1980.4	0.03751	1.401	Homo sapiens neurogranin (protein kinase C substrate, RC3) (NRGN), mRNA.
*LOC100133583*	1081.4	870.6	0.03818	0.805	PREDICTED: Homo sapiens similar to major histocompatibility complex, class II, DQ beta 1, transcript variant 2 (LOC100133583), mRNA.
*GABARAPL1*	536.9	417.1	0.03826	0.776	Homo sapiens GABA(A) receptor-associated protein like 1 (GABARAPL1), mRNA.
*PTGFRN*	13.2	24	0.03829	1.818	Homo sapiens prostaglandin F2 receptor negative regulator (PTGFRN), mRNA.
*PDXK*	436.9	514.6	0.0385	1.177	Homo sapiens pyridoxal (pyridoxine, vitamin B6) kinase (PDXK), mRNA.
*ATG2A*	339.2	287.2	0.0385	0.846	Homo sapiens ATG2 autophagy related 2 homolog A (S. cerevisiae) (ATG2A), mRNA.
*TAGAP*	330.2	259	0.0393	0.784	Homo sapiens T-cell activation RhoGTPase activating protein (TAGAP), transcript variant 2, mRNA.
*NAT8B*	127.4	184.4	0.03936	1.447	Homo sapiens N-acetyltransferase 8B (GCN5-related, putative, gene/pseudogene) (NAT8B), mRNA.
*C19orf33*	23.3	42.5	0.0396	1.824	Homo sapiens chromosome 19 open reading frame 33 (C19orf33), mRNA.
*MYLIP*	1777.6	1479.2	0.03961	0.832	Homo sapiens myosin regulatory light chain interacting protein (MYLIP), mRNA.
*ANKRD27*	113.7	93.1	0.03962	0.818	Homo sapiens ankyrin repeat domain 27 (VPS9 domain) (ANKRD27), mRNA.
*PATL1*	853.9	731.7	0.03985	0.856	Homo sapiens protein associated with topoisomerase II homolog 1 (yeast) (PATL1), mRNA.
*MCL1*	1439.3	1263.9	0.03995	0.878	Homo sapiens myeloid cell leukemia sequence 1 (BCL2-related) (MCL1), transcript variant 1, mRNA.
*LOC643733*	107.4	87.4	0.04028	0.813	PREDICTED: Homo sapiens hypothetical LOC643733 (LOC643733), mRNA.
*S1PR5*	811.2	591	0.04044	0.728	Homo sapiens sphingosine-1-phosphate receptor 5 (S1PR5), mRNA.
*GNLY*	3203.7	2308.7	0.04079	0.720	Homo sapiens granulysin (GNLY), transcript variant NKG5, mRNA.
*C17orf56*	96.3	77.7	0.04085	0.806	Homo sapiens chromosome 17 open reading frame 56 (C17orf56), mRNA.
*PCNT*	594.2	500.2	0.04122	0.841	Homo sapiens pericentrin (PCNT), mRNA.
*GFI1*	217.8	168.2	0.04128	0.772	Homo sapiens growth factor independent 1 transcription repressor (GFI1), mRNA.
*GLTPD1*	36.1	48.6	0.04149	1.346	Homo sapiens glycolipid transfer protein domain containing 1 (GLTPD1), mRNA.
*SLC25A4*	154.7	126.6	0.04161	0.818	Homo sapiens solute carrier family 25 (mitochondrial carrier; adenine nucleotide translocator), member 4 (SLC25A4), nuclear gene encoding mitochondrial protein, mRNA.
*HBEGF*	839.1	550.7	0.0417	0.656	Homo sapiens heparin-binding EGF-like growth factor (HBEGF), mRNA.
*FAM179A*	128.1	91.4	0.04173	0.713	Homo sapiens family with sequence similarity 179, member A (FAM179A), mRNA.
*TNFAIP3*	2436.9	1817.1	0.04194	0.745	Homo sapiens tumor necrosis factor, alpha-induced protein 3 (TNFAIP3), mRNA.
*GBP2*	3234.4	2664.8	0.04267	0.823	Homo sapiens guanylate binding protein 2, interferon-inducible (GBP2), mRNA.
*TRIM11*	186.6	156.9	0.04292	0.840	Homo sapiens tripartite motif-containing 11 (TRIM11), mRNA.
*MAFB*	3522	2710.3	0.04307	0.769	Homo sapiens v-maf musculoaponeurotic fibrosarcoma oncogene homolog B (avian) (MAFB), mRNA.
*CEBPA*	755.7	907.2	0.04315	1.200	Homo sapiens CCAAT/enhancer binding protein (C/EBP), alpha (CEBPA), mRNA.
*GBP1*	592.5	334.1	0.0434	0.563	Homo sapiens guanylate binding protein 1, interferon-inducible, 67kDa (GBP1), mRNA.
*FANCG*	154.3	128.9	0.04358	0.835	Homo sapiens Fanconi anemia, complementation group G (FANCG), mRNA.
*C1orf166*	215.1	181.7	0.04383	0.844	Homo sapiens chromosome 1 open reading frame 166 (C1orf166), mRNA.
*OTUD1*	563.3	444.8	0.04392	0.789	PREDICTED: Homo sapiens OTU domain containing 1 (OTUD1), mRNA.
*RNASE6*	712.7	850.7	0.04399	1.193	Homo sapiens ribonuclease, RNase A family, k6 (RNASE6), mRNA.
*IL18R1*	326.3	274.9	0.04408	0.842	Homo sapiens interleukin 18 receptor 1 (IL18R1), mRNA.
*FAM82A2*	661.2	567.8	0.04481	0.858	Homo sapiens family with sequence similarity 82, member A2 (FAM82A2), mRNA.
*ITPRIP*	823.8	701	0.04497	0.850	Homo sapiens inositol 1,4,5-triphosphate receptor interacting protein (ITPRIP), mRNA.
*TM6SF1*	158.5	196.8	0.04506	1.241	Homo sapiens transmembrane 6 superfamily member 1 (TM6SF1), mRNA.
*SH2D2A*	111.2	91.5	0.04507	0.822	Homo sapiens SH2 domain protein 2A (SH2D2A), mRNA.
*TBC1D8*	164.6	133.6	0.04512	0.811	Homo sapiens TBC1 domain family, member 8 (with GRAM domain) (TBC1D8), mRNA.
*KBTBD2*	780.8	668.5	0.04523	0.856	Homo sapiens kelch repeat and BTB (POZ) domain containing 2 (KBTBD2), mRNA.
*LOC728835*	1550.2	788.3	0.04542	0.508	PREDICTED: Homo sapiens similar to cytokine, transcript variant 3 (LOC728835), mRNA.
*DDX60*	282.4	208.8	0.04543	0.739	Homo sapiens DEAD (Asp-Glu-Ala-Asp) box polypeptide 60 (DDX60), mRNA.
*HLA-G*	1282.7	1105.5	0.04544	0.861	Homo sapiens HLA-G histocompatibility antigen, class I, G (HLA-G), mRNA.
*TXNDC11*	263.6	223.9	0.04555	0.849	Homo sapiens thioredoxin domain containing 11 (TXNDC11), mRNA.
*SYTL1*	359.6	307	0.04565	0.853	Homo sapiens synaptotagmin-like 1 (SYTL1), mRNA.
*CCL4L1*	1781.5	845.5	0.04659	0.474	Homo sapiens chemokine (C-C motif) ligand 4-like 1 (CCL4L1), mRNA.
*PARP12*	374.2	299.3	0.04667	0.799	Homo sapiens poly (ADP-ribose) polymerase family, member 12 (PARP12), mRNA.
*ACTN1*	1248.3	1617.6	0.04701	1.295	Homo sapiens actinin, alpha 1 (ACTN1), mRNA.
*TST*	683.6	805.1	0.04719	1.177	Homo sapiens thiosulfate sulfurtransferase (rhodanese) (TST), nuclear gene encoding mitochondrial protein, mRNA.
*ZNF366*	8.7	17.7	0.04733	2.034	Homo sapiens zinc finger protein 366 (ZNF366), mRNA.
*CCL2*	335.1	178.2	0.04737	0.531	Homo sapiens chemokine (C-C motif) ligand 2 (CCL2), mRNA.
*RNF10*	415.4	350.3	0.04757	0.843	Homo sapiens ring finger protein 10 (RNF10), mRNA.
*FCER1A*	1459.3	1873.7	0.04808	1.283	Homo sapiens Fc fragment of IgE, high affinity I, receptor for; alpha polypeptide (FCER1A), mRNA.
*IFI44L*	662.6	264.8	0.04823	0.399	Homo sapiens interferon-induced protein 44-like (IFI44L), mRNA.
*C1orf177*	26.2	37.2	0.04823	1.419	Homo sapiens chromosome 1 open reading frame 177 (C1orf177), mRNA.
*OAS3*	435.9	272.1	0.04838	0.624	Homo sapiens 2'-5'-oligoadenylate synthetase 3, 100kDa (OAS3), mRNA.
*LOC728006*	140.1	170.1	0.04866	1.214	PREDICTED: Homo sapiens hypothetical protein LOC728006 (LOC728006), mRNA.
*ATP6V0A1*	489.6	577.4	0.04873	1.179	Homo sapiens ATPase, H+ transporting, lysosomal V0 subunit a1 (ATP6V0A1), transcript variant 3, mRNA.
*SLC2A1*	494.2	421.3	0.04882	0.852	Homo sapiens solute carrier family 2 (facilitated glucose transporter), member 1 (SLC2A1), mRNA.
*SPOCD1*	23.6	42.4	0.04902	1.796	Homo sapiens SPOC domain containing 1 (SPOCD1), mRNA.
*IDI1*	435.2	348.6	0.04916	0.801	Homo sapiens isopentenyl-diphosphate delta isomerase 1 (IDI1), mRNA.
*SERPINF1*	56.9	40.1	0.04927	0.704	Homo sapiens serpin peptidase inhibitor, clade F (alpha-2 antiplasmin, pigment epithelium derived factor), member 1 (SERPINF1), mRNA.
*CDC45L*	32	18.4	0.04934	0.575	Homo sapiens CDC45 cell division cycle 45-like (S. cerevisiae) (CDC45L), mRNA.
*KLHDC5*	203.5	247.7	0.04958	1.217	Homo sapiens kelch domain containing 5 (KLHDC5), mRNA.
*KLRG1*	407.4	294.4	0.0497	0.722	Homo sapiens killer cell lectin-like receptor subfamily G, member 1 (KLRG1), mRNA.
*LOC728276*	-1.5	6.5	0.04973	4.333	PREDICTED: Homo sapiens similar to Lithostathine 1 precursor (Pancreatic stone protein 1) (PSP) (Pancreatic thread protein 1) (PTP) (Islet of Langerhans regenerating protein 1) (REG 1) (LOC728276), mRNA.

AVG–average. Fold change values lower than 1 represent down-regulation of the gene in the high anhedonia group compared to low anhedonia group, while values higher than 1 represent up-regulation.

**Fig 1:**
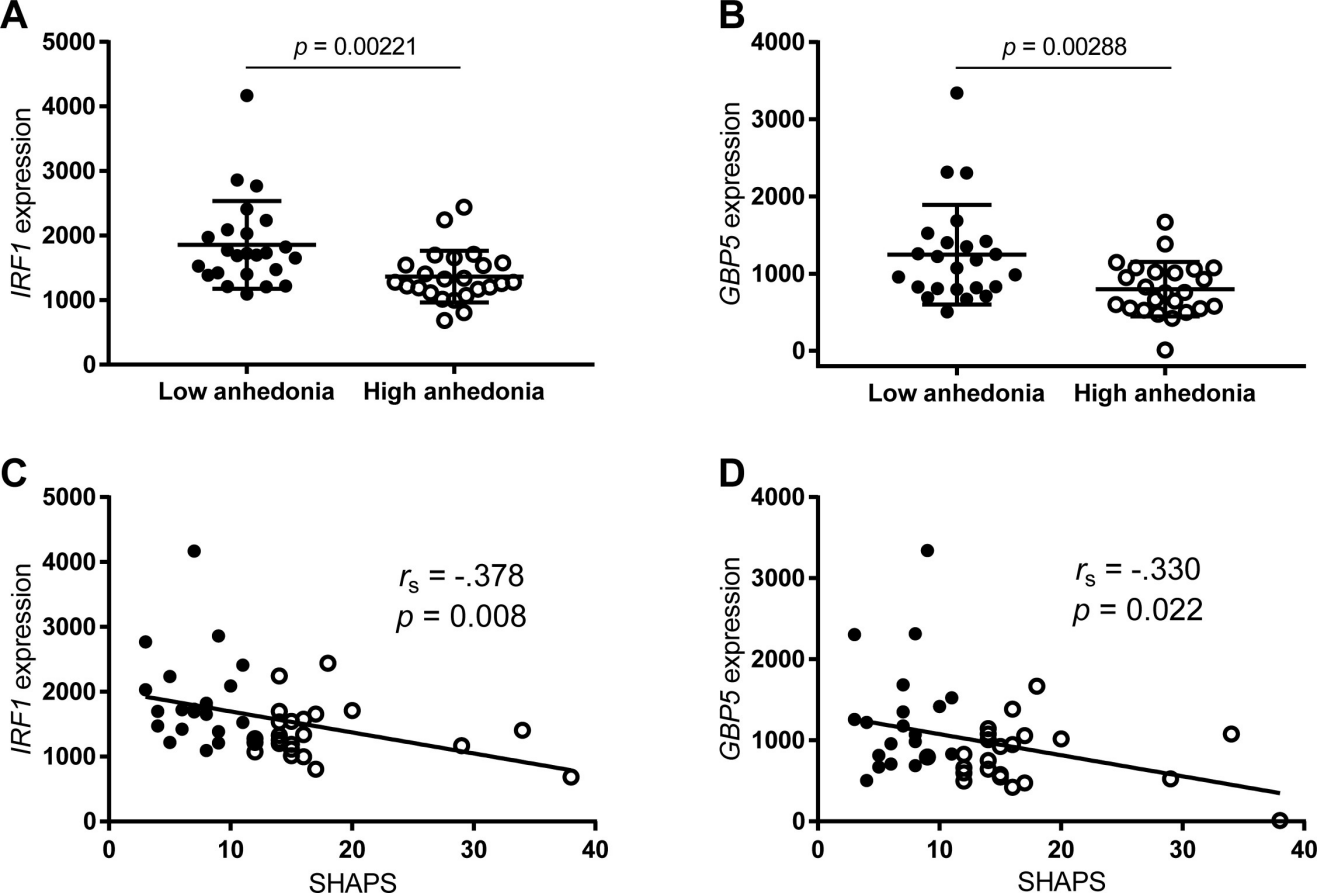
Gene expression of interferon regulatory factor 1 (IRF1) and guanylate binding protein 5 (GBP5) in peripheral blood mononuclear cells from patients with cocaine use disorder with low and high symptoms of anhedonia. A and B) Between-group comparison of *IRF1* (A) and *GBP5* (B) expression values. Dots represent individual values for each subject and lines represent mean ± standard deviation. Comparisons were made with Mann-Whitney U tests. C and D) Spearman’s rank-order correlation between total Snaith-Hamilton Pleasure Scale (SHAPS) scores and the expression of *IRF1* (C) and *GBP5* (D).

The top differentially expressed genes were *IRF1* (*p* = 0.00221) and *GBP5* (*p* = 0.00288), which were both significantly downregulated in the high anhedonia group (Fig 1A and 1B). Significant correlations were found between microarray expression of both genes and the total SHAPS score (*IRF1* –*r*_*s*_ = -0.378, *p* = 0.008; *GBP5* –*r*_*s*_ = -0.330, *p* = 0.022, Fig 1C and 1D). Findings from the microarray were partly validated by qPCR, with measures of *IRF1* and *GBP5* obtained from both methods showing significant correlations (S1 Fig). However, the relationships between these individual genes and anhedonia did not sustain statistical significance when gene expression was measured by qPCR data, although trends were similar (*IRF1* –Mann-Whitney U = 210, *p* = 0.108; *GBP5* –U = 197, *p* = 0.061; S2 Fig).

Pathway analysis performed on DAVID showed that the 166 differentially expressed genes were enriched for mechanisms related to defense response to virus, antiviral defense, immunity, interferon-gamma-mediated signaling pathway, among others (Table 3). Functional annotation clustering analysis was also performed to reduce the redundancy of our findings and confirmed mechanisms related to immunity as the top-relevant pathways associated with anhedonia in our sample (S1 Table).

**Table 3:** Pathway analysis (functional annotation) of the nominally differentially expressed genes (n = 166) between cocaine use disorder patients with low and high anhedonia.

Category	Term	Count	%	P-value	FDR
GOTERM_BP_DIRECT	defense response to virus	13	9,6	2,1E-9	3,2E-6
UP_KEYWORDS	Antiviral defense	11	8,1	3,8E-9	4,8E-6
UP_KEYWORDS	Immunity	18	13,3	2,2E-8	2,8E-5
GOTERM_BP_DIRECT	interferon-gamma-mediated signaling pathway	8	5,9	6,9E-7	1,1E-3
GOTERM_BP_DIRECT	immune response	15	11,1	1,7E-6	2,6E-3
GOTERM_BP_DIRECT	type I interferon signaling pathway	7	5,2	6,0E-6	9,3E-3
UP_KEYWORDS	Innate immunity	11	8,1	7,3E-6	9,1E-3

Gene-term enrichment performed on DAVID v. 6.8 (https://david.ncifcrf.gov/). The most relevant (overrepresented) biological terms associated with the list of differentially expressed genes were identified by the DAVID functional annotation chart. Enrichments were calculated with a modified Fisher’s exact test controlled for false discovery rate (FDR).

## Discussion

In this study, we examined genome-wide gene expression in peripheral blood mononuclear cells, with the hypothesis that participants with CUD and high levels of anhedonia would display a unique expression of inflammatory pathways, compared to individuals with CUD and low anhedonic symptoms. No individual genes were determined to be significantly different between the low and high anhedonia groups using stringent controls for false discovery rate. However, an exploratory analysis to identify differentially expressed genes with nominal *p* < 0.05 found 166 putative *loci* that differed between groups. DAVID, a bioinformatics tool that provides functional interpretations of complex lists of genes, was utilized to examine representation of these top differentially expressed genes in known gene pathways. It confirmed that mechanisms related to immunity were the most pertinent pathways corresponding with anhedonia in our sample. The two top differentially expressed genes in our sample were chosen for additional validation. These were *IRF1* and *GBP5*; genes related to transcriptional regulation, tumor response, inflammation, and innate immunity. Expression of both genes significantly correlated with total scores on our self-report of anhedonia, further substantiating the relationship of these gene expression changes to anhedonia.

Several studies have linked cocaine use to inflammatory mechanisms [15,33]. However, our results suggest that some CUD patients display greater changes in inflammatory markers than others, suggesting a more complex and heterogeneous association between CUD and inflammation. In particular, these alterations in immune functioning were associated with a poor prognostic sign for CUD, anhedonia. Based on these results, it seems possible that a subset of patients with CUD present a pro-inflammatory genetic profile that interacts with cocaine use or other associated environmental factors to promote an anhedonic (and treatment-resistant) phenotype. Although our groups did not differ on basic demographics or measures of drug use, these changes may also be reflective of subtler differences in intensity or frequency of cocaine use, unmeasured environmental or psychosocial factors, biological and genetic differences, or gene by environment interactions, as seen with other genetic variants in addiction [34,35]. Our study is the first to provide preliminary evidence for the hypothesis that anhedonia in CUD is related to inflammatory changes in gene-expression, but a more in-depth assessment in a larger sample size that could include examination of potential gene *vs*. environment interactions is warranted.

As hypothesized, the top-ranked pathways associated with the differentially expressed genes in our sample were all linked to inflammatory mechanisms. Further, both top differentially expressed genes have immune-related functions. *IRF1* serves as a transcriptional activator of several genes involved in both innate and acquired immune responses, as well as in tissue response to inflammation, cell proliferation, and programmed cell death [36–38]. The relationship of *IRF1* to cocaine use is unknown, but it could plausibly be modulated by dopamine-enhancing properties of cocaine and the cellular signaling pathways that are activated by both D1- and D2-like dopamine receptors after cocaine use [39]. Dopamine receptor D1 is known to activate protein kinase A (PKA), which in turn can serine-phosphorylate IRF1 [40]. Once phosphorylated, IRF1 has been shown to activate IFN-α/β promoters to induce their endogenous expressions [41–43], ultimately affecting the immune/inflammatory response [44]. Moreover, cocaine use has been associated with alterations in the levels of leptin [45], which is an adipokine produced by both the adipose tissue and immune cells and participates in innate and adaptive immunity [46]. Interestingly, leptin has been shown to promote the activation and recruitment of a transcriptional complex between IRF1 and CREB [47], suggesting another layer of regulation by which cocaine could interfere with IRF-1 mechanisms and ultimately regulate inflammation. Similarly, *GBP5*’s expression is not only induced by IFN-γ [48], but is also an activator of the NLRP3 inflammasome [49], with key roles in innate immunity and overall inflammation [50]. There is no reported evidence of a direct effect of cocaine or the activation of dopamine receptors on the expression or function of GBP5, limiting the further interpretation of the relationship of this factor to cocaine use. Our study is the first to report altered levels of these gene transcripts both in CUD and in relation to anhedonia. Both genes were found to be downregulated in high anhedonia CUD patients compared to those with low anhedonia, suggesting a dysfunction in inflammatory mechanisms in these patients. Interestingly, IRF-1 is also downregulated by stress-related hormones [51], consistent with hypotheses that drug use and stress produce similar and synergistic effects on immune responses [52], and hypotheses linking excessive inflammatory responses to stress with anhedonia [53]. Further, one study found that chronic methamphetamine administration in mice resulted in downregulation of GBP-5 expression in the nucleus accumbens. Together these findings support our results by suggesting that although the identification of these transcripts in CUD is novel, they are affected in consistent directions by other stressors, including other stimulant drugs.

There has been increasing recognition of the value of using genome-wide transcription profiles to drive medication development in addiction, with examples in both alcohol use disorder [54] and methamphetamine use disorder [55]. Although our top gene candidates are not part of the “druggable genome” that can be directly targeted with already-developed compounds, our results nevertheless suggest critical future directions for CUD treatment research. Specifically, medication development efforts in CUD have often focused on agonist strategies using stimulant medications [56], but these medications may themselves have inflammatory effects, resulting in poor outcomes for individuals with an anhedonic/inflammatory profile. Our research suggests that prioritizing anti-inflammatory medications in development for stimulant use disorders, such as pioglitazone [57] and ibudilast [58], may pay dividends, particularly in combination with treatment-matching strategies using either the anhedonic phenotype or biomarkers of inflammatory gene expression to personalize treatment. Interestingly, a recent study found that an improvement in the hedonic tone induced by repetitive transcranial magnetic stimulation (rTMS) in CUD patients was associated with a reduction in the craving for cocaine [59]. A future direction for research may be to explore whether these improvements are associated with inflammatory alterations, and whether the inflammatory genes identified in our study may be able to predict outcomes in treatments like this that are directed at ameliorating anhedonia in CUD patients.

Our results need to be interpreted in light of some limitations. First, the relationship between peripheral and central changes in gene expression and/or inflammation is ultimately unknown. Although research suggests some substantive correspondences between peripheral and central gene expression [23], our top candidates are not among those transcripts with confirmed peripheral/central relationships. It is also the case that we do not know the degree to which the observed mRNA alterations relate to altered production of inflammatory compounds in the brain or periphery. Future studies should focus on the correlation of our identified inflammatory markers between blood and brain to allow a further discussion and more accurate interpretation of our findings, especially as they may relate to neuroinflammation or systemic inflammation. Although prior studies suggest a relationship between *increased* inflammation and anhedonia, we found *downregulation* of two genes generally presumed to be pro-inflammatory. In the absence of more direct measures of the state of either peripheral or central immune functioning in these participants, these results do not allow us to directly infer the presence of inflammation, only that immune functioning appears to differ between our groups. There are also limitations inherent in measuring genome-wide expression via a microarray, which is restricted to the probes available in the Beadchip and may not capture the full snapshot of gene expression alterations in CUD [60]. In addition, our analysis did not allow for the detection of specific transcripts derived from the same gene, which might have masked important information regarding alternative splicing mechanisms and their potential involvement in anhedonia [23]. A network-based approach assessing the interaction between several genes and transcripts detected by next-generation sequencing techniques might provide more comprehensive and biologically-relevant results in the future. In this sense, to actually confirm our microarray results and explore their full clinical implications, the findings obtained in this analysis (which were only partially replicated by qPCR) require further biological validation by the measurement of protein levels, the targeted assessment of proteins belonging to the same signaling pathways, and replication in independent cohorts. We also did not test for differences in blood cell type composition between the two groups [61], which, if present, could also confound our results to some extent. In addition, we included some patients presenting with highly typical medical and psychiatric comorbidities of CUD in our groups, including those with hypertension, high cholesterol, and non-psychotic psychiatric disorders such as depression and PTSD in our analysis. All of these conditions are thought to be associated with chronic inflammation [62,63]. While we carefully matched the groups on these potential confounders, we did not have the sample size needed to statistically rule out that they may have influenced our results. We are also not able to establish a direction of causality for relationships between anhedonia, CUD and inflammation–in the absence of either longitudinal data or temperament measures of anhedonia that are assumed to be more stable (e.g. [64] we cannot establish whether anhedonia precedes or results from either CUD or inflammation. Another limitation is that our small sample size was not powered to yield FDR-corrected results, raising the possibility type I errors. Although we attempted to protect against this to some extent by taking a bioinformatics approach that tested the likelihood of a *pattern* of changes appearing, use of larger sample sizes in follow-up studies will be important for replication and validation of our preliminary findings, as well as for allowing a statistical correction for the potential confounding clinical variables described above. Likewise, future analyses would significantly benefit from the inclusion of a healthy control group, allowing for further interpretations of the size of gene expression changes between high and low anhedonia groups and the analysis of the potential interaction between the CUD diagnosis and anhedonia. Last, as discussed earlier, integration of these analyses with genotype data (e.g. by expression quantitative trait *loci* analyses) will be particularly important to assess for the involvement of genotypic differences.

## Conclusions

In summary, we found that the poor prognostic symptom of anhedonia in CUD is associated with changes in peripheral gene expression in pathways related to inflammatory mechanisms and immune response. This is consistent with evidence from other psychiatric disorders suggesting that anhedonia is related to inflammation, and suggests that anti-inflammatory strategies should be explored in treatment-resistant CUD. Our results form part of an exciting new body of research that uses genome-wide changes in expression to highlight novel directions for treatment research, identifying mechanisms that may not have been a focus of past treatment development efforts.

## Supporting information

S1 Fig Real-time quantitative PCR (qPCR) validation of microarray results. Both *IRF1* (A) and *GBP5* (B) show statistically significant correlations between both methods (Spearman’s rank order correlation tests) in our sample of cocaine use disorder patients with low (n = 24) and high anhedonia (n = 24).
(TIF)

S2 Fig Gene expression of interferon regulatory factor 1 (IRF1) and guanylate binding protein 5 (GBP5) measured by quantitative real-time PCR in peripheral blood mononuclear cells from patients with cocaine use disorder with low and high symptoms of anhedonia. A and B) Between-group comparison of *IRF1* (A) and *GBP5* (B) expression values. Dots represent individual values (normalized for the expression of beta-2-microglobulin (*B2M*) and calculated by the delta delta Ct method) for each subject and lines represent mean ± standard deviation. Comparisons were made with Mann-Whitney U tests. C and D) Spearman’s rank-order correlation between total Snaith-Hamilton Pleasure Scale (SHAPS) scores and the expression of *IRF1* (C) and *GBP5* (D) measured by quantitative real-time PCR.
(TIFF)

S1 Table Functional annotation clustering. (DOCX)
