# Supplementary figures and images for: Anhedonia in cocaine use disorder is associated with inflammatory gene expression
Source: PLoS One. 2018 Nov 8;13(11):e0207231. doi: 10.1371/journal.pone.0207231 (PMC6224118; doi:10.1371/journal.pone.0207231)

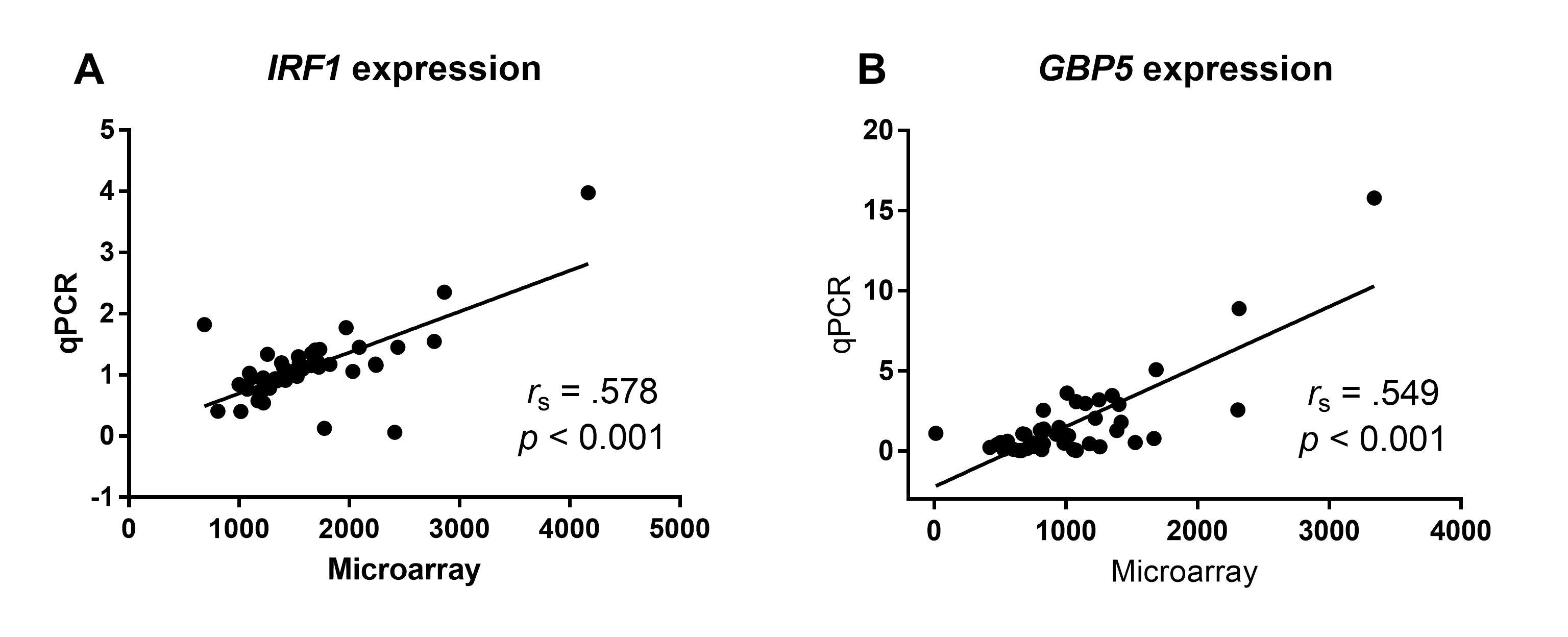

Supplement: S1 Fig — Both IRF1 (A) and GBP5 (B) show statistically significant correlations between both methods (Spearman’s rank order correlation tests) in our sample of cocaine use disorder patients with low (n = 24) and high anhedonia (n = 24). (TIF) [file pone.0207231.s001.tif]

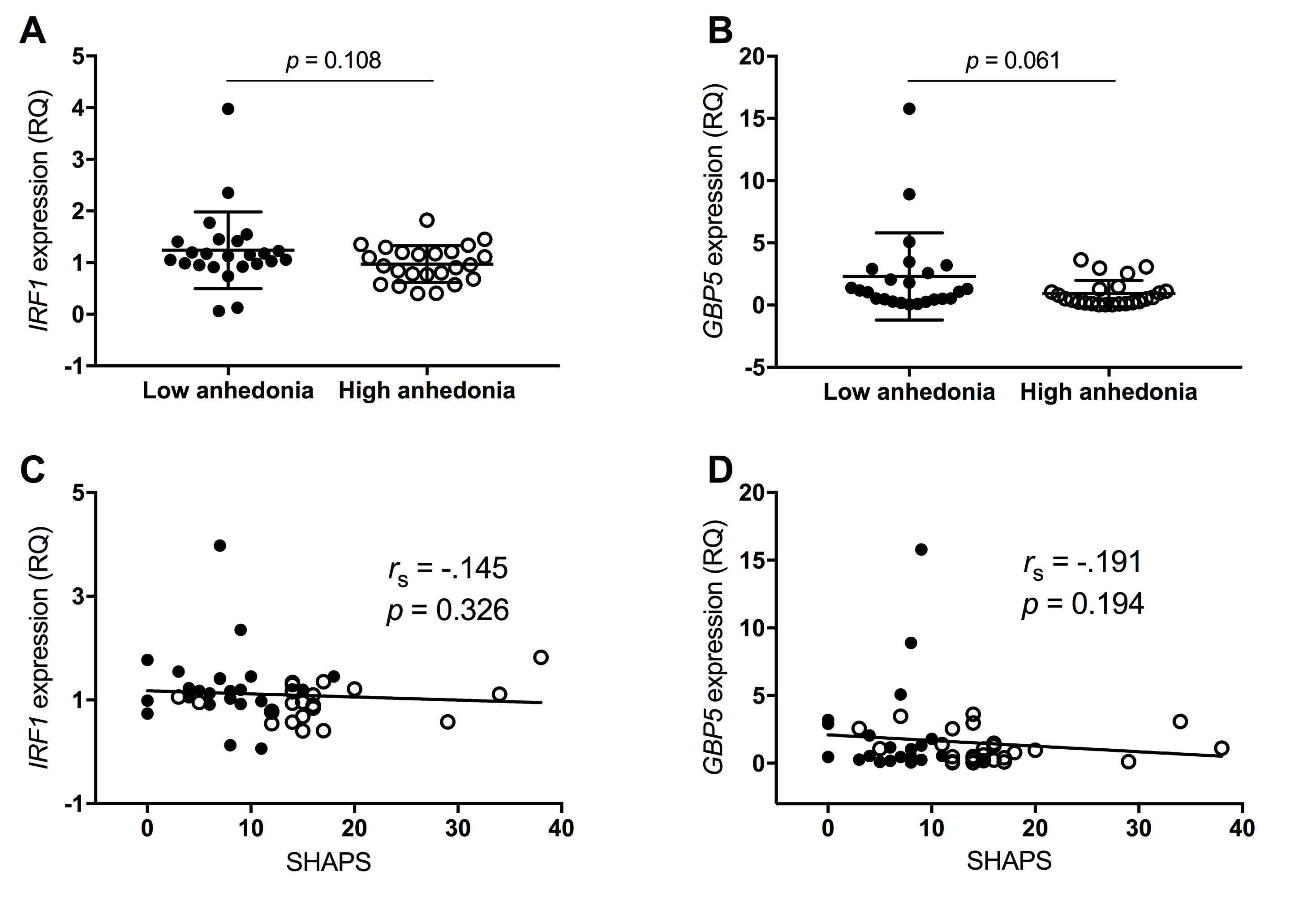

Supplement: S2 Fig — A and B) Between-group comparison of IRF1 (A) and GBP5 (B) expression values. Dots represent individual values (normalized for the expression of beta-2-microglobulin (B2M) and calculated by the delta delta Ct method) for each subject and lines represent mean ± standard deviation. Comparisons were made with Mann-Whitney U tests. C and D) Spearman’s rank-order correlation between total Snaith-Hamilton Pleasure Scale (SHAPS) scores and the expression of IRF1 (C) and GBP5 (D) measured by quantitative real-time PCR. (TIFF) [file pone.0207231.s002.tiff]
