# Supplementary material for: Anhedonia in cocaine use disorder is associated with inflammatory gene expression
Source: PLoS One. 2018 Nov 8;13(11):e0207231. doi: 10.1371/journal.pone.0207231 (PMC6224118; doi:10.1371/journal.pone.0207231)
Supplement: S1 Table — (DOCX) [file pone.0207231.s003.docx]

**S1 Table**. **Functional annotation clustering**

| **Category** | **Term** | **Count (%)** | **Genes** | **FDR** |  |
| --- | --- | --- | --- | --- | --- |
| **Annotation Cluster 1** | | | **Enrichment score: 6.272876** | | |
| GOTERM_BP_DIRECT | GO:0051607: defense response to virus | 13 (9.6) | *CD8A, IFITM3, OAS3, HERC5, IFI44L, APOBEC3H, STAT1, TRIM22, TRIM11, NLRC5, DDX60,* ***IRF1****, GBP1* | 3.21E-06 |  |
| UP_KEYWORDS | Antiviral defense | 11 (8.1%) | *DDX60, IFITM3, OAS3, HERC5,* ***IRF1****, IFI44L, APOBEC3H, STAT1, TRIM22, GBP1, TRIM11* | 4.81E-06 |  |
| UP_KEYWORDS | Immunity | 18 (13.3) | *TNFRSF21,* ***GBP5****, CD8A, IFITM3, FFAR2, OAS3, HERC5, APOBEC3H, SLAMF7, HLA-G, NLRC5, CD55, KLRG1, DDX60, TAP1,* ***IRF1****, RIPK2, GBP1* | 2.81E-05 |  |
| UP_KEYWORDS | Innate immunity | 11 (8.1) | *NLRC5, CD55, KLRG1, DDX60, IFITM3, OAS3, HERC5,* ***IRF1****, RIPK2, SLAMF7, APOBEC3H* | 0.009139213 |  |
| **Annotation Cluster 2** | | | **Enrichment score: 3.987926** | | |
| GOTERM_BP_DIRECT | GO:0060333: interferon-gamma-mediated signaling pathway | 8 (5.9) | *OAS3,* ***IRF1****, IRF4, STAT1, TRIM22, GBP2, HLA-G, GBP1* | 0.001064158 |  |
| GOTERM_BP_DIRECT | GO:0060337: type I interferon signaling pathway | 7 (5.1) | *IFITM3, OAS3,* ***IRF1****, IRF4, STAT1, GBP2, HLA-G* | 0.009268874 |  |

Legend: Functional annotation clustering analysis performed on DAVID v. 6.8 (https://david.ncifcrf.gov/). Enrichment score of the cluster is the minus log-transformed geometric mean of all the enrichment *P*-values of each annotation term in the group. Highlighted genes (*IRF1* and *GBP5*) are the two top-ranked differentially expressed genes between groups validated by quantitative real-time PCR. FDR – false discovery *q*-value.
